# Cleaning by clustering: methodology for addressing data quality issues in biomedical metadata

**DOI:** 10.1186/s12859-017-1832-4

**Published:** 2017-09-18

**Authors:** Wei Hu, Amrapali Zaveri, Honglei Qiu, Michel Dumontier

**Affiliations:** 1 State Key Laboratory for Novel Software Technology, Nanjing University, 163 Xianlin Avenue, Nanjing, 210023 Jiangsu China; 2 Institute of Data Science, Maastricht University, Maastricht, 6200 MD The Netherlands

**Keywords:** GEO, Metadata, Data quality, Clustering, Biomedical, Experimental data, Reusability

## Abstract

**Background:**

The ability to efficiently search and filter datasets depends on access to high quality metadata. While most biomedical repositories require data submitters to provide a minimal set of metadata, some such as the Gene Expression Omnibus (GEO) allows users to specify additional metadata in the form of textual key-value pairs (e.g. sex: female). However, since there is no structured vocabulary to guide the submitter regarding the metadata terms to use, consequently, the 44,000,000+ key-value pairs in GEO suffer from numerous quality issues including redundancy, heterogeneity, inconsistency, and incompleteness. Such issues hinder the ability of scientists to hone in on datasets that meet their requirements and point to a need for accurate, structured and complete description of the data.

**Methods:**

In this study, we propose a clustering-based approach to address data quality issues in biomedical, specifically gene expression, metadata. First, we present three different kinds of similarity measures to compare metadata keys. Second, we design a scalable agglomerative clustering algorithm to cluster similar keys together.

**Results:**

Our agglomerative cluster algorithm identified metadata keys that were similar, based on (i) name, (ii) core concept and (iii) value similarities, to each other and grouped them together. We evaluated our method using a manually created gold standard in which 359 keys were grouped into 27 clusters based on six types of characteristics: (i) age, (ii) cell line, (iii) disease, (iv) strain, (v) tissue and (vi) treatment. As a result, the algorithm generated 18 clusters containing 355 keys (four clusters with only one key were excluded). In the 18 clusters, there were keys that were identified correctly to be related to that cluster, but there were 13 keys which were not related to that cluster. We compared our approach with four other published methods. Our approach significantly outperformed them for most metadata keys and achieved the best average F-Score (0.63).

**Conclusion:**

Our algorithm identified keys that were similar to each other and grouped them together. Our intuition that underpins cleaning by clustering is that, dividing keys into different clusters resolves the scalability issues for data observation and cleaning, and keys in the same cluster with duplicates and errors can easily be found. Our algorithm can also be applied to other biomedical data types.

## Background

Enormous amounts of biomedical data have been and are being produced at an unprecedented rate by researchers all over the world. However, in order to enable reuse, there is an urgent need to understand the structure of datasets, the experimental conditions under which they were produced and the information that other investigators may need to make sense of the data [1]. That is, there is a need for accurate, structured and complete description of the data — defined as *metadata*.

Gene Expression Omnibus (GEO) is one of the largest, best-known biomedical databases [2]. GEO is an international public repository for high-throughput microarray and next-generation sequence functional genomic data submitted by the research community. As of 2016, the GEO database hosts >69,000 public series (study records) submitted directly by over 3000 laboratories, comprising over 1,800,000 “Samples” and over 44,000,000 sample characteristics captured as unconstrained key-value pairs^1^. Users submit data to GEO via a spreadsheet (namely *GEOarchive spreadsheet*), which requires them to fill out a metadata template that follows the guidelines set out by the Minimum Information About a Microarray Experiment (MIAME) guidelines [3]. The metadata template includes fields for title, summary, overall design, contributors, protocols (e.g. growth, treatment, extraction, labeling, hybridization, scanning, and data processing) as well as sample characteristics (e.g. sex, organism, tissue, cell type). After submission, a curator checks the content and validity of the information provided [4]. This process is not only time-consuming but also error-prone considering the amount of manual labor that is involved. GEO allows users to specify additional metadata in the form of textual key-value pairs (e.g. sex: female). However, since there is no structured vocabulary to guide the submitter regarding the metadata terms to use, consequently, the 44,000,000+ key-value pairs in GEO suffer from numerous quality issues. Moreover, without a standardized set of terms with which to fill out the template fields, there are different versions of the same entity without any (semantic) links between them. Thus, we chose GEO as a use case for our study.

As it currently stands, GEO metadata suffers from several quality issues including redundancy, heterogeneity, inconsistency, incompleteness, etc. These key-value pairs are manually entered by the submitters and have different spellings (e.g. age: 21 years, age_yrs: 21) or use different terms to define the same concept (e.g. disease: Still, illness: Still). For instance, the key “age” itself has over 31 different variants. Specifically for the key “age in years”, there are heterogeneous notations such as “age (y)”, “age in years”, age (years)”, “age at diagnosis (years)” or “age (yrs)”. On the other hand, for biomedical concepts such as the key “disease” has different notations such as“ disease”, “illness”, “clinical type”, “infection status” or “healthy control”, which are lexically very different thus making it hard to identify similar keys. Additionally, corresponding to these keys are a myriad of values, heterogeneous in themselves such as different notations of the same disease name or the value of age. Thus, when one attempts to find similar studies by querying the metadata using keywords (as available by the GEO website), all the related studies are not retrieved, resulting in loss of important information. Thus, as a first step, we aim to identify and resolve such quality issues in the *keys* of the millions of GEO Sample records.

Good quality metadata is essential in finding, interpreting, and reusing existing data beyond what the original investigators envisioned. This, in turn, can facilitate a data-driven approach by combining and analyzing similar data to uncover novel insights or even more subtle trends in the data. These insights can then be formed into hypothesis that can be tested in the laboratory [2]. Thus, scalable methodologies to curate the existing metadata, which is of poor quality, is of prime importance to help enable reuse of the vast amounts of valuable biomedical data. Poor metadata quality has important implications for the re-usability of data. In [5], the authors performed a multi-cohort analysis of the publicly available gene expression datasets, which revealed a robust diagnostic signature for sepsis. To execute their study, the authors were forced to use a variety of keywords to retrieve a large set of potential datasets and subsequently examine each one to identify essential metadata and ensure that they met their inclusion criteria. Such laborious approaches pose a critical barrier in scaling up their approach so as to find diagnostic signatures for other disorders.

Thus, we propose cutCluster, an algorithm for scalable agglomerative clustering to group similar keys together so as to identify the closely-related ones as well as the erroneous ones in order to tackle the metadata quality problem, specifically for gene expression data. Our intuition that underpins cleaning by clustering is that, dividing keys into different clusters resolves the scalability issues for data observation and cleaning, and keys in the same cluster with duplicates and errors can easily be found. Related work includes Freudenberg et al. [6] who developed a computational framework for analytically and visually integrating knowledge-base functional categories with the cluster analysis of genomics data, based on the gene-specific functional coherence scores. Loureiro et al. [7] describes a methodology of the application of hierarchical clustering methods to the task of detecting erroneous foreign trade transactions. Ulrich et al. [8] provided an R implementation for the affinity propagation clustering technique, which has gained increasing popularity in bioinformatics. For concept matching, Giunchiglia et al. [9] presented basic and optimized algorithms for semantic matching and discussed their implementation within the S-Match system. Using clustering to data cleaning is widely accepted in practice to improve data quality, and our clustering algorithm incorporates various similarity measures and is very scalable for cleaning gene expression metadata.

## Methods

**Fig. 1:**
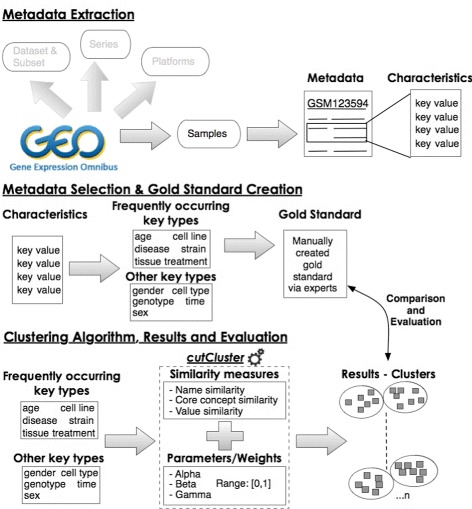
Steps undertaken for applying our cutCluster algorithm to perform cleaning by clustering of the GEO metadata, i.e., characteristics keys

In this section, we explain the extraction and selection process of the GEO dataset metadata, particularly the keys, as the first step since it was unknown how many different key categories are present. This was followed by the gold standard creation on a subset of the keys (since one did not already exist) to validate our approach. Then, we present details of our three similarity measures and cutCluster, our clustering algorithm, used for the clustering of the selected GEO keys. Figure 1 displays our proposed workflow including the specific steps undertaken in the process.

### Dataset metadata extraction

**Fig. 2:**
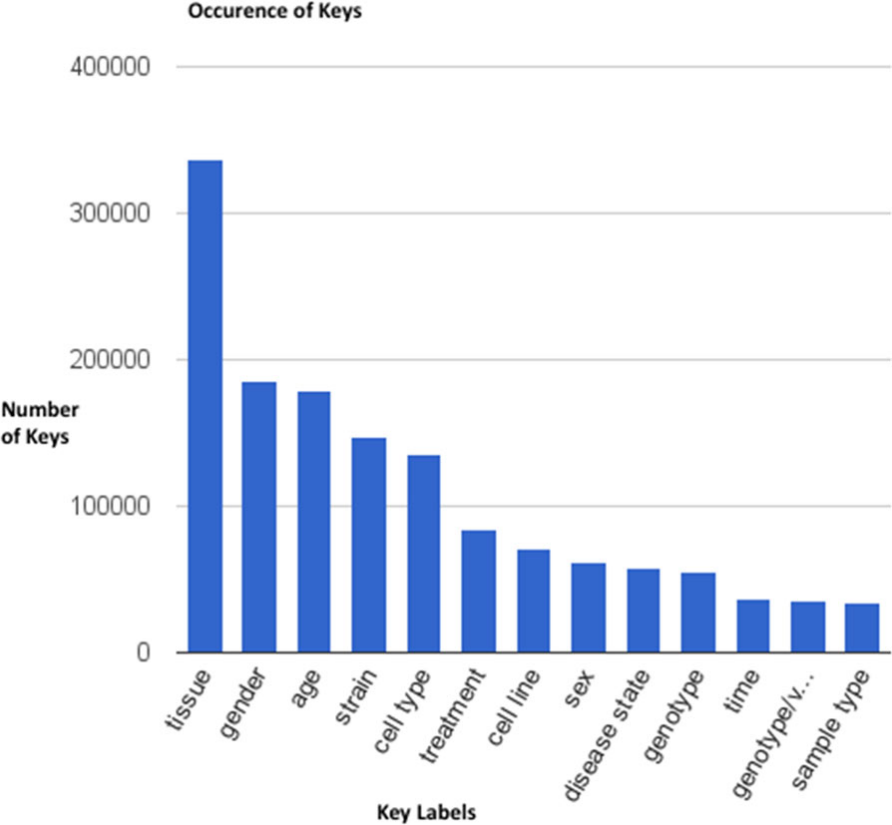
Number of occurrences of the top 20 occurring keys in GEO

As our use case, we selected metadata from the GEO dataset, in particular, from the “Sample” records. A Sample record describes the conditions under which an individual Sample was handled, the manipulations it underwent, and the abundance measurement of each element derived from it. In a Sample, from these different metadata elements, we specifically chose the “Characteristics” field (see Fig. 1), which contains information about, for example, tissue, age, gender, cell type, disease and strain, used in the study. This information is structured in the key-value pair format. For example, in the Sample GSM549324, one of the key-value pair is gender: Male, where “gender” is the key and “Male” is the value. In the entire GEO dataset, there are over 44,000,000 key-value pairs. Figure 2 shows the occurrence of the top 20 keys in GEO.

As a first step, we aim to identify and resolve such quality issues in the *keys* of the GEO Sample records. The problems in the keys range from (i) minor spelling discrepancies (e.g. age at diagnosis (years), age at diagonosis (years); genotype/varaiation, genotype/varat, genotype/varation, genotype/variaion, genotype/variataion genotype/variation), (ii) having different syntactic representations (e.g. age (years), age(yrs) and age_year), (iii) using different terms altogether to denote one concept (e.g. disease vs. illness vs. healthy control) or (iv) using two different key terms in one (e.g. disease/cell type, tissue/cell line, treatment age).

Thus, when one attempts to find similar studies by querying the metadata using keywords (as available at the GEO website), some related studies would not be retrieved resulting in loss of important information. We used the SQLite3 GEO database^2^ to acquire the GEO metadata. We then retrieved a sample of these GEO keys and created a gold standard for them, as described in the next section.

### Metadata selection and gold standard creation

Out of over 11,000 unique keys in GEO, to test our approach, we chose 359 keys. That is, we queried the dataset using regular expressions with a key string to retrieve all the different variants of that key. We first chose six key categories, namely (i) age, (ii) cell line, (iii) disease, (iv) strain, (v) tissue and (vi) treatment, as these are the most frequently occurring ones (c.f. Figs. 1 and 2). In order to validate our results and since one did not already exist, we created a gold standard of all these keys by manually dividing these 359 keys into several clusters. In total, we created 31 reference clusters, where four clusters with only one key were excluded. The remaining 27 clusters with 355 keys were considered as the gold standard. The average number of keys in each cluster was 13, and the standard deviation was 13.84. The maximum and minimum numbers of keys in a cluster were 78 and 3, respectively. This gold standard is available at http://ws.nju.edu.cn/geo-clustering/. Our next step was to perform the clustering based on three similarity measures as explained in the next section.

### Similarity measures

To resolve various heterogeneities in the GEO keys, we explored three types of similarities to compare any two GEO keys (see Fig. 1):

- *Name similarity*, denoted by *sim*_*name*_(), is computed by comparing the lexical names of the keys, such as “tissue isolated” and “tissue derived”.
- *Core concept similarity*, denoted by *sim*_*core*_(), is computed by comparing the most important concepts (called *core concepts* [10]) in the names of the keys. The core concept is either the first verb in the name that is greater than four characters long or, if there is no such verb, the first noun in the name, together with any adjective modifying that noun. For example, the core concept of “tissue isolated” is “isolated”, while the core concept of “tissue derived” is “derived”. We first extracted the core concepts using Stanford NLP parser [11] and then extended these concepts with synonyms obtained from a thesauri http://www.thesaurus.com.
- *Value similarity*, denoted by *sim*_*value*_(), is calculated by comparing all the values, e.g. “Male”, “Female”, of a key, e.g. “gender”. We chose the highest score from the similarities of all value pairs.

To measure the similarities between strings, we used the Jaro-Winkler method, since it repeatedly performs well, among the best, for tasks like ontology alignment [12] and record matching [13].

To formalize, given two keys *t*_*i*_,*t*_*j*_, the overall similarity, denoted by *sim*(), between *t*_*i*_,*t*_*j*_ is defined as a weighted combination of the name, core concept and value similarities:

1 $$\begin{array}{*{20}l} sim(t_{i},t_{j})=&\ \alpha\cdot sim_{name}(t_{i},t_{j}) \\ &+\beta\cdot sim_{core}(t_{i},t_{j}) \\ &+\gamma\cdot sim_{value}(t_{i},t_{j}), \end{array} $$

where *α*,*β*,*γ* are the weighting factors in [0,1] range, s.t. *α*+*β*+*γ*=1. We used a linear regression to train the weights for the combination. More details are provided in the “Discussion” section.

### cutCluster – our clustering algorithm

The goal of cutCluster, our clustering algorithm, is to categorize a set of keys into a set of disjoint clusters, denoted by *C*_1_,*C*_2_,…,*C*_*n*_, whereby some measure, the cohesion between the keys in a cluster *C*_*i*_ is high, meanwhile the coupling across different clusters *C*_*i*_,*C*_*j*_ is low. Following the conventional definition of clustering, we assumed that all clusters together equals the complete set of keys, and any two different clusters are mutually disjoint. Our intuition that underpins cleaning by clustering is that, dividing keys into different clusters resolves the scalability issues for data observation and cleaning, and keys in the same cluster with duplicates and errors can easily be found.

We re-designed the agglomerative (bottom-up) clustering algorithm [14, 15], which is a scalable hierarchical clustering algorithm for large ontology matching. The pseudo code of the cutCluster is depicted in Algorithm 1, which accepts as input a set of keys and returns a set of clusters. Initially, it establishes a singleton cluster for each key, and sets its cohesion equal to 1 (Line 6). The coupling between any two keys is set to their overall similarity (Line 8). During each iteration, it selects the cluster set **C** ^∗^ with the greatest cohesion (Line 12), and finds the cluster pair (*C*_*s*_,*C*_*t*_) with the greatest coupling (Line 13). After merging *C*_*s*_ and *C*_*t*_ into a new cluster *C*_*p*_ (Line 19), it updates the cohesion of *C*_*p*_ as well as its coupling with other ones (Lines 20–22). The time complexity of this algorithm is *O*(|**T**|^2^), where **T** denotes the set of keys.



As compared with the previous algorithm [15], the new termination condition depends on the threshold of coupling rather than the maximum number of keys in a cluster. Another main difference is that the distance measure proposed in this paper is based on linguistic similarities, while [15] leveraged structural proximities (which are difficult to calculate here due to the plain hierarchy between the keys).

For the criterion function, we proposed *cut*() to calculate both cohesion and coupling, which measures the cutting cost of two clusters by considering the aggregated inter-connectivity of them. Formally, let *C*_*i*_,*C*_*j*_ be two clusters. The cutting cost, denoted by *cut*(), of *C*_*i*_,*C*_*j*_ is defined as follows:

2 $$\begin{array}{*{20}l} cut(C_{i},C_{j})=\frac{\sum\limits_{t_{i}\in C_{i}}\sum\limits_{t_{j}\in C_{j}}sim(t_{i},t_{j})}{|C_{i}|\cdot|C_{j}|}, \end{array} $$

where *sim*() represents the overall similarity measure in Eq. (1) and | | counts the number of keys in a cluster. When *C*_*i*_,*C*_*j*_ refer to the same cluster, *cut*() calculates the cohesion of this cluster, i.e., *cohesion*(*C*_*i*_)=*cut*(*C*_*i*_,*C*_*i*_); Otherwise, it computes the coupling between them, i.e., *coupling*(*C*_*i*_,*C*_*j*_)=*cut*(*C*_*i*_,*C*_*j*_). Using this uniform criterion function simplified our clustering algorithm and made those previously-calculated distances reusable in the next iterations.

**Fig. 3:**
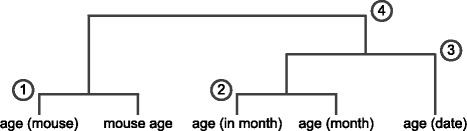
Running example showing the different variants of the key “age”

**Running example** To help understand, we show a running example in Fig. 3. Given five keys involving “age” in a dataset, “age (mouse)”, “mouse age”, “age (in month)”, “age (month)” and “age (date)”, the dendogram of our clustering result is depicted in the figure. Specifically, “age (in month)” and “age (month)” are likely to be duplicates, and month is related to date in some sense according to www.thesaurus.com.

## Results

In this section, we present the clustering results and their interpretations and with the evaluation along with the metrics.

### Results

By using cutCluster, our agglomerative clustering algorithm and setting the coupling threshold *ε* to 0.5, 18 clusters were generated, containing all the 355 keys in the gold standard. The average number of keys in a cluster is 20, and the standard deviation is 20.34. The maximum and minimum numbers of keys in a cluster are 78 and 3, respectively. All the results are available on our website and listed in Table 1.

**Table 1:** Clustering results on six keys: (i) age, (ii) cell line, (iii) disease, (iv) strain, (v) tissue and (vi) treatment with the number of keys

No. of keys	Key
	**Age**
25	Age unit, age group, age_years, age (y), age in years, donor_age, age (months), age (years), age (yrs), patient age, age at diagnosis, age at diagnosis (years), age at sample (months), patient age (yrs), tumor stage, age.brain, age (weeks), stage, gestational age (weeks), age.blood, sample age, age at surgery, age, age months, age(years)
5	Pathological_stage, growth/development stage, growth stage, pathological stage, development stage
	**Cell line**
12	Cell line name, cell line source age, cell line type, cell lines, cell line background, cell lineage, cell line/clone, cell line source gender, cell line source ethnicity, cell line, cell line passage, cell line source
3	Origin of a cell line, source cell line, growth pattern of cell line
14	Tissue/cell line, cell line source tissue, dendritic cell lineages, coriell cell line repository identifier, cell line tissue source, parental cell line, tumor cell line, donor cell line, tissue/cell lines, injected cell line, tumour cell line used for conditioning medium, insect cell line, cell line origin, primary cell line
	**Disease**
5	Subject’s disease state, primary disease, histology (disease state), advanced disease stage, advanced disease state
22	Disease-state, meibomian gland disease state, disease, disease/treatment status, disease status of patient, disease progression, disease stage, disease subtype, status of disease, clinical characteristic/disease status, patient disease status, disease development, disease phase, diseased, disease/cell type, extent of disease, disease state, disease state (host), disease severity, disease_state, disease model, disease type
7	Disease_specific_survival_years, disease status, diseasestatus, disease_specific_survival_event, disease outcome, disease exposure, disease_status
16	Disease-free survival (dfs), disease-free interval (months), disease free interval (days), disease specific survival (years), stage of disease (inss), disease relapse (event), disease_free_survival_event, disease-free survival (dfs) event, disease_free_survival_years, disease progression (event), stage of disease, disease free interval (months), age at disease onset, duration of disease, disease free survival in months, disease free survival time (months)
	**Strain**
22	Background mouse strain, background/strain, background strains, strain, strain/accession, strain or line, strain/background, strain/genotype, strain/ecotype, strains, strain number, strain [background], strain phenotype, strain/line, strain description, strain source, strain fgsc number, strain background (bloomington stock number), strain (donor)
3	Toxoplasma parasite strain, infection (virus strain), human cytomegalovirus strain
16	Bacteria strain, siv strain, viral strain, recipient strain, substrain, parent strain, parental strain, host strain, parasite strain, host strain background, maternal strain, virus strain, scanstrain, mice strain, mouse strain, plant strain
	**Tissue**
14	Sample tissue of origin, cell line source tissue, cell/tissue type, original tissue, source tissue, cell line, tissue source, organ/tissue, original tissue source, primary tissue, sample tissue type, sample type, cell tissue, source tissue type, organ/tissue type
3	Age of ffpe tissue, day of tissue dissection, age at tissue collection (days)
78*	Tissue separation, tissue & age, tissuer type, tissue_detail, tumor tissue source, tissue/tumor subtype, tissue derivation, tissue, tissue origination, tissue site, tissue_mg, tissue/cell lines, tumor/tissue type, tissue subtype, tissue_biological, tissue processing, tissue/development stage, harvested tissue type, tissue and developmental stage, tissue isolated
	**Treatment**
67*	Pretreatment drug & dose, pre-treatment, treatment2_in vivo treatment, treatment stage, treatments, treatment agent, treatment_molecule, lighttreatment, drug treatment time point, treatment result, treatment_2, treatment_1, tissue treatment, cactus host treatment, inducer treatment, sirna treatment group, treatment/exposure, maternal treatment group, treatment_dose, treatment dosage
12	l-dopa treatment, patient treatment plan, nrti treatment status, culture conditions/treatment, tamoxifen-citrate treatment, disease/treatment status, globin treatment, experimental treatment, dopamine-agonists treatment, oxygen treatment, tap treatment, lenolidamide treatment
31	Time of treatment, treatment time, tissue/treatment id, treatment period, days after treatment, treatment duration, pre-treatment psa, treatment time (rhgaa), weeks of treatment, tnfa treatment time point, treatment_time, treatment length, time (days post-treatment), order of treatment, bl treatment level, treatment-time, time after treatment, day of dss treatment, time post treatment, time of tamoxifen treatment, h2o2 treatment level, days of ddc treatment, weeks after treatment, post-treatment time, length of treatment, duration of il-6 treatment, treatment start age, duration of treatment, days of treatment, time post-treatment, treatment age

^*^Due to space constraints, only the first 20 keys are reported in this table for the “age” cluster with 78 keys and the “treatment” cluster with 67 keys, respectively. All results are available on our website

**Fig. 4:**
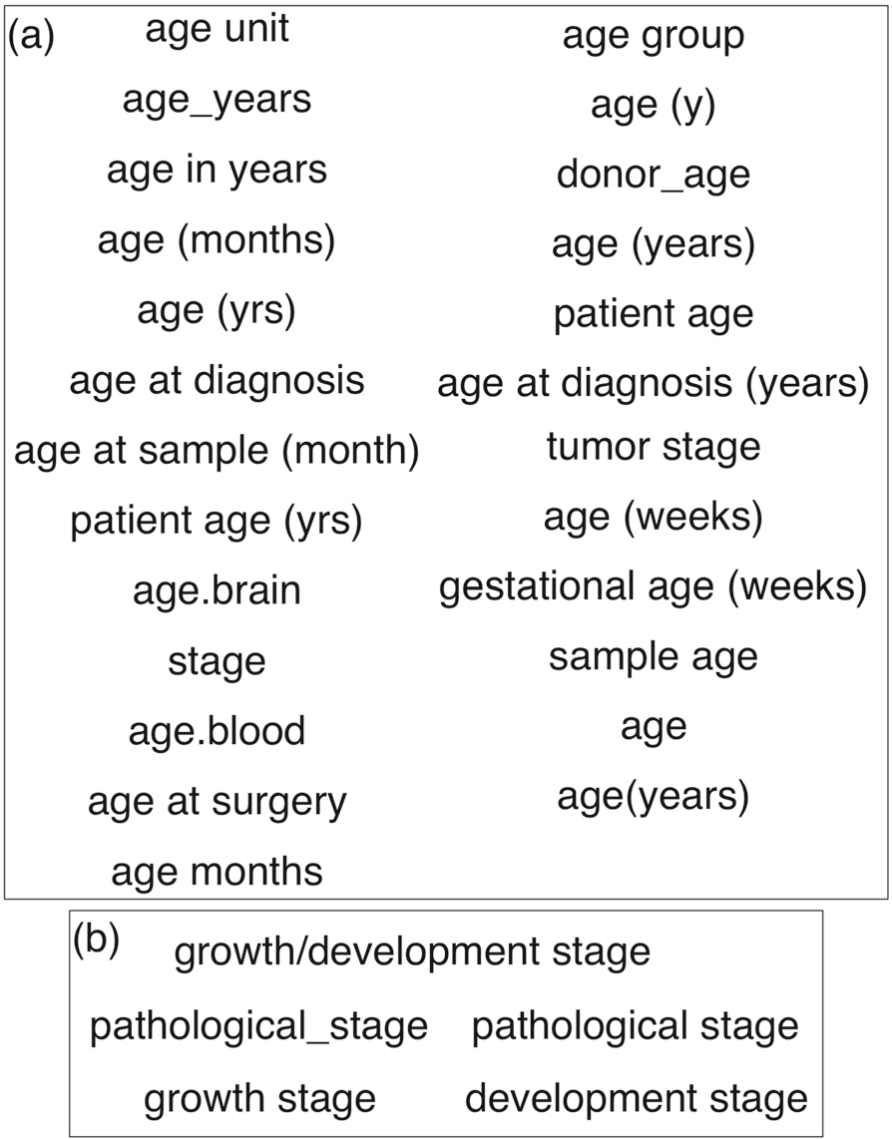
Two clusters for the key “age”, see panels (**a**) and (**b**), respectively

Upon further analysis of the clusters themselves, we found that there were keys that were identified correctly to be related to that particular clusters, but there were keys which were incorrectly clustered as they belong to another key cluster or in some cases belong to more than one cluster. For example, the two clusters for the characteristic key “age” are depicted in Fig. 4. On one hand, in Fig. 4 a, “age (years)”, “age (months)” and “age (weeks)” were clustered together correctly. On the other hand, in Fig. 4 b, “growth stage”, “development stage” and “pathological stage” are clustered together, which do not belong correctly to the “age” cluster but are classified in this cluster due to the stem “age” occurring in the keys.

**Fig. 5:**
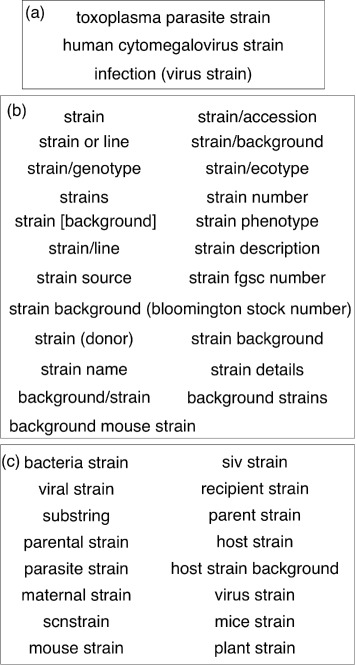
Three clusters for the key “strain”, see panels (**a**), (**b**) and (**c**), respectively

Similarly, for the key “strain” (as depicted in Fig. 5), there were three clusters containing 3, 22 and 16 keys respectively. In Fig. 5 a, the keys “toxoplasma parasite strain”, “human cytomegalovirus strain” and “infection (virus strain)” were correctly clustered together as they are all related to virus strains. In Fig. 5 b, the keys related to “strain” were clustered together. Additionally, “bacteria mouse strain”, “background/strain” and “background strains” were group together where “bacteria mouse strain” did not belong to the cluster but was included due to the stem “bac” in it, which was matched to “back” from the other two keys. In Fig. 5 c, the keys related to bacterial, parasite or virus strains were correctly clustered together. However, it was difficult to determine which cluster the key “strain/cell line background” best belonged to as the value was a PubMed ID.

For the “cell line” cluster, there were 4 keys that were incorrectly grouped into these clusters: “cell line source age”, “cell line source tissue”, “cell line source gender” as they belonged to another cluster namely, “age”, “tissue” and “gender” respectively. However, for the key “cell line/genotype” with the value “by4741 (wt)”, it was unclear which cluster this key best belonged to.

For the “disease” keys cluster, there were 18 keys that were incorrectly grouped into this cluster as they belonged to the “time” category (e.g. “disease free interval (days)”, “disease-free interval (months)”, “disease duration (yrs)”). However, for the key “code disease-specific survival” with the values “0” and “1”, it was unclear which cluster this key best belonged to.

For the “age” keys cluster, the keys indicating a stage (e.g. “growth stage”, “tissue stage”, “lyme disease stage”) were incorrectly grouped in the “age” cluster but belonged to the “time” cluster as their values indicated a time point. The key “8 weeks. tissue” belonged to the “tissue” cluster and the key “sexual maturity” belonged to the “gender” cluster. Keys that belonged to more than one cluster were: (i) “age and tissue”, which belonged to both “age” and “tissue” and (ii) “age(years)/gender”, (iii) “age/sex”, (iv) “age/gender”, (v) “gender and age”, which belonged to both “age” and “gender” clusters.

For the “tissue” keys category, there were keys that belonged to the “time” cluster (e.g. “# of tissue = 36 tissue”, “age of ffpe tissue”, “day of tissue dissection”, “8 weeks. tissue”). Additionally, there were keys that belonged to the “genotype”, “cell type” clusters (e.g. “tissue genotype/variation”, “tissue/cell line”). However, “tissue/treatment id” could also belong to the “treatment” group. But, since the values of this key were 4, 3, 2, it was difficult to determine the best fit.

For the “treatment” keys category, there were 23 keys whose values denoted a time point (e.g. “length of treatment (days)”, “treatment stage”, “age (at the end of treatment)”) and thus belonged to the “time” cluster.

From our analysis, we observed that even though we are able to correctly detect keys and their variants, which belong to one cluster (key type), there are cases which require human verification (e.g. via crowdsourcing) to choose the best fit by analyzing the values.

### Evaluation

**Metrics** We chose three well-known metrics for clustering evaluation [16]: the *F-Score*, denoted by *FS*(), the *entropy*, denoted by *E*(), and the *Rand index*, denoted by *RI*(), to assess the algorithmic clusters against the reference ones (i.e. gold standard) that were manually built beforehand.

For calculating the first two metrics, two operators, namely *precision* and *recall*, denoted by *P*() and *R*() respectively, were employed to compare a cluster with another. Formally, given the computed cluster set **C** and the reference cluster set **R**, let *C*_*i*_ be a computed cluster in **C** (1≤*i*≤*N*), and *R*_*j*_ be a reference cluster in **R** (1≤*j*≤*M*). *C*_*i*_∩*R*_*j*_ computes the common keys shared by *C*_*i*_ and *R*_*j*_, while | | counts the number of keys in a cluster. The precision and recall of *C*_*i*_ w.r.t. *R*_*j*_ are defined as follows:

3 $$\begin{array}{*{20}l} P(C_{i},R_{j}) &= \frac{|C_{i}\cap R_{j}|}{|C_{i}|}, \end{array} $$

4 $$\begin{array}{*{20}l} R(C_{i},R_{j}) &= \frac{|C_{i}\cap R_{j}|}{|R_{j}|}. \end{array} $$

The clustering-based F-Score is defined as the combination of precision and recall, whose value is in [0,1] range, and a higher value indicates a better clustering quality. The clustering-based F-Score of **C** w.r.t. **R** is defined as follows:

5 $$\begin{array}{*{20}l} FS\left(\mathbf{C},\mathbf{R}\right)&=\frac{\sum_{i=1}^{N} FS\left(C_{i},\mathbf{R}\right)\cdot|C_{i}|}{\sum_{i=1}^{N}|C_{i}|}, \end{array} $$

6 $$\begin{array}{*{20}l} FS\left(C_{i},\mathbf{R}\right)&=\max_{1\leq j\leq M}\frac{2\cdot P(C_{i},R_{j})\cdot R(C_{i},R_{j})}{P(C_{i},R_{j})+R(C_{i},R_{j})}. \end{array} $$

The entropy measures the distribution of keys between clusters and indicates the overall clustering quality. A lower entropy value implies a better clustering quality. The best possible entropy value is 0, while the worst is 1. An alternative metric based on the information theory is *NMI* (Normalized Mutual Information). Given the computed cluster set **C** and the reference cluster set **R**, the entropy of **C** w.r.t. **R** is defined as follows:

7 $$\begin{array}{*{20}l} E(\mathbf{C},\mathbf{R})&=\frac{\sum_{i=1}^{N} E(C_{i},\mathbf{R})\cdot|C_{i}|}{\sum_{i=1}^{N} |C_{i}|}, \end{array} $$

8 $$\begin{array}{*{20}l} E(C_{i},\mathbf{R})&=-\frac{\sum_{j=1}^{M} P(C_{i},R_{j})\cdot\log P(C_{i},R_{j})}{\log M}. \end{array} $$

The Rand index measures the similarity between two clustering results by penalizing both false positive and false negative decisions. The value of Rand index is in [0,1], and a higher value indicates a better clustering quality. The Rand index of **C** w.r.t. **R** is defined as follows:

9 $$\begin{array}{*{20}l} RI(\mathbf{C},\mathbf{R})=\frac{TP+TN}{\binom{|\mathbf{T}|}{2}}, \end{array} $$

where *TP* denotes the number of key pairs that are in the same cluster in **C** and in the same cluster in **R**, while *TN* denotes the number of key pairs that are in different clusters in **C** and in different clusters in **R**. **T** denotes the set of keys in **R**.

**Comparative clustering algorithms.** We selected four off-the-shelf clustering algorithms for comparison. We briefly describe them as follows:

- K-medoid [17] is a partition clustering algorithm related to K-means, with the differences of choosing “real” data points as centers (called *medoids*), and working with an arbitrary metric of distances between data points.
- DBSCAN [18] is one of the most common density-based clustering algorithm, which groups together points that are closely packed, marking as outliers points that stay alone in low-density regions.
- APCluster [8] allows for determining typical cluster members (called *exemplars*), and applies affinity propagation to exemplar-based agglomerative clustering, which has gained increasing popularity in bioinformatics.
- StdHier represents the standard hierarchical clustering algorithm implemented in clusterMaker—a multi-algorithm clustering plug-in for Cytoscape [19]. Cytoscape implements the Standard Hierarchical clustering in Java, in which the average-linkage method is used [20].

We re-implemented the K-medoid and DBSCAN algorithms, and tuned parameters to obtain best performance. K-medoid got two clusters for “age” and “treatment”, three clusters for “cell line” and “disease”, six clusters for “strain” and 13 clusters for “tissue”. For DBSCAN, *eps* was tuned from 0 to 1 step by 0.01, while *minPts* was tuned from 0 to 100 step by 1. Parameters were varied of different keys. For StdHier and DBSCAN, we used (1−similarity) as their distance function to calculate the distance between any two terms. We adopted default parameters of APCluster and StdHier which were implemented in the clustering plug-in for Cytoscape.

Table 2 shows the comparison results between our agglomerative clustering algorithm, cutCluster, and the four other comparative algorithms. From this table, we can see that our algorithm significantly outperformed the other algorithms in most characteristic keys (except “cell line”) and achieved the best average F-Score (0.63), entropy (0.58) and Rand index (0.64), which demonstrate better consistency between our algorithm and human experts.

**Table 2:** Comparison on F-Score (*FS*), Entropy (*E*) and Rand Index (*RI*)

Key (ref. cluster number)	Weights			Our algorithm			K-medoid			DBSCAN			APCluster			StdHier		
	*α*	*β*	*γ*	*FS*	*E*	*RI*	*FS*	*E*	*RI*	*FS*	*E*	*RI*	*FS*	*E*	*RI*	*FS*	*E*	*RI*
Age (2)	.44	.01	.55	**.94**	**.34**	.**87**	.86	.51	.67	.87	.43	.69	.68	.59	.54	.81	.60	.63
Cell line (4)	.65	.11	.24	.46	.78	.56	**.60**	.78	.54	.49	.78	.40	.59	**.70**	**.64**	.52	.82	.43
Disease (4)	.15	.18	.67	.58	**.55**	**.65**	.64	.58	.61	.63	.69	.36	**.67**	.63	.52	.61	.58	.63
Strain (4)	.85	.00	.15	**.58**	.69	**.62**	.43	.68	.61	.50	.76	.35	.42	.68	.46	.48	.78	.35
Tissue (9)	.80	.00	.20	.43	.73	.37	.41	.69	.56	**.49**	.77	.27	.35	.74	**.58**	.40	**.68**	.45
Treatment (4)	.57	.00	.43	.78	**.41**	**.74**	.69	.58	.67	.76	.69	.47	.68	.69	.50	**.81**	.58	.66
Average				**.63**	**.58**	**.64**	.61	.64	.61	.62	.69	.42	.57	.67	.54	.60	.67	.52

A higher F-Score, a higher Rand Index or a lower entropy indicates a better quality, and the best ones are formatted as bold

**Fig. 6:**
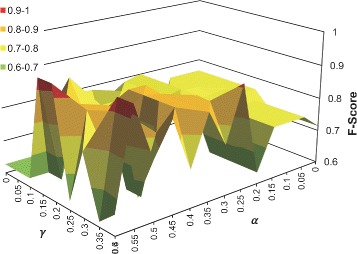
Change of cutCluster’s performance w.r.t. different *α*,*β*,*γ* values

Additionally, Table 2 shows the weights of *α*,*β* and *γ* for achieving the best similarity combination, which varies between the characteristic keys. Due to the small amount of the keys involving each characteristic key, we did not conduct *n*-fold cross-validation in this evaluation. Table 3 shows the F-Scores of comparing the result of clustering each characteristic key separately with the clustering of all keys as a whole dataset. Note that it is inappropriate to compare them using entropy or Rand index, because these two measures are dominantly affected by the number of clusters, e.g. *M* in Eq. (8) and *TN* in Eq. (9). From the table, we observe that the F-Scores are much better if the characteristic keys are separated, because a unified set of parameters is not suitable for different keys, especially when the numbers of keys in different clusters are highly imbalanced. This verifies the effectiveness of our workflow by first dividing dataset into small keyword categories using keywords and regular expressions, and then conducting clustering on each category. Figure 6 shows the change of performance with respect to different *α*, *β* and *γ* values. Note that *α* + *β* + *γ* = 1. The figure shows the different F-Scores for the “age” category. We can see that the actual performance for a range of weighting factors is not far from the best. For the other five categories (Table 2), we observed similar results, which indicated that, although we cannot achieve the best result by clustering the whole dataset, there is a range of choices that make the result acceptable on each keyword category in practice (also demonstrated by Table 3). That is, although a gold standard may not always be available, there are still many choices that can be made to achieve a good result. Our empirical experience is that, the weights for name and value similarities (*α* and *γ*, respectively) are broadly effective, while the weight for core concept similarity (*β*) depends on features of the characteristic keys.

**Table 3:** F-Score (*FS*) comparison between dividing the dataset based on characteristic keywords and taking it as a whole

	cutCluster	K-medoid	DBSCAN	APCluster	StdHier
Average [Min, Max]	0.63 [0.43, 0.94]	0.61 [0.41, 0.86]	0.62 [0.49, 0.87]	0.57 [0.35, 0.68]	0.6 [0.4, 0.81]
As a whole dataset	0.43	0.4	0.37	0.34	0.4

**Application to other most frequent keys** We applied our agglomerative clustering algorithm on five characteristic keys that had the highest frequency, excluding the ones that have already been evaluated in Table 2. Our clustering results are shown in Table 4, which demonstrate the feasibility of our algorithm on various large-scale data.

**Table 4:** Clustering results on other most frequent keys

Keys	Key frequency	Cluster number	Max.	Min.	Avg.
			Key number per cluster		
Gender	188,277	4	17	2	11
Cell type	137,192	5	14	1	6
Genotype	100,876	5	28	20	22
Time	100,462	14	241	3	29
Sex	67,529	4	16	4	8

Key frequency denotes the number of key-value pairs that include that particular key

**Fig. 7:**
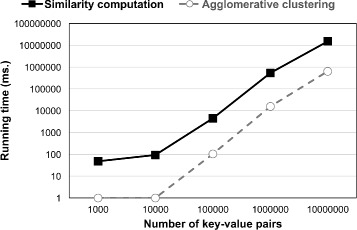
Simulation depicting scalability of the similarity computation and agglomerative clustering

**Scalability** In order to determine the scalability of our method, we simulated the performance as depicted in Fig. 7. The simulation was performed on a personal workstation with an Intel Xeon E3 3.2 GHz CPU and 16 GB memory. We observe that our similarity computation and agglomerative clustering can both deal with large-scale datasets.

## Discussion

The clustering results that we have presented allow us to make the following observations about cutCluster’s strengths and weaknesses. By looking at the generated clusters in detail, we found that hierarchical clustering is suitable for biomedical metadata cleaning. That is, it helps in clustering keys which are similar to one another. Let us take the key category “disease” as an example. The keys “disease free interval (months)” and “disease-free interval (months)” were grouped together, which are probably duplicates of each other and thus can be easily identified. Furthermore, our agglomerative clustering algorithm can make clusters at different granularities. For instance, “disease free interval” and “disease free survival” were assigned in the same cluster at a higher layer, but separated into different clusters at a lower layer of the tree (in the case of hierarchical clustering).

Our agglomerative clustering algorithm followed a bottom-up approach and preferred to merge smaller clusters into larger ones. However, we found that it did not perform very well on skewed clustering, which means that some clusters possessed a large amount of keys while the others had few. For example, the numbers of keys in the two gold standard clusters for key cell line are 32 and 5 respectively. Furthermore, we compared our algorithm with four representative competitors, but there exist numerous hierarchical algorithms, thus it is hard, if not impossible, to compare all of them for biomedical metadata cleaning.

We selected the threshold by referencing the gold standard built by human experts. However, it is difficult to know an appropriate clustering granularity without a gold standard. Moreover, the weights for combining name, core concept and value similarities varied between characteristic keys, and we have not found an optimal method to automatically determine them to achieve the best clustering quality. It is worth noting that creating reference clusters as gold standard is a time-consuming and subjective process. This is why we designed our workflow (Fig. 1) such that we first divided the datasets into smaller chunks (by selecting keys using keywords and regular expressions) and performed clustering on each part. We manually created the gold standard on this small part to validate our approach and then apply cutCluster to another set of keys, where no gold standard is present. The experimental results demonstrated that this workflow can improve accuracy. Specifically, it shows the strength of our approach in using hierarchical clustering as a means to cluster similar keys and enabling the user to choose the level at which the clusters are best formed.

Additionally, unlike evaluating ontology/schema mappings using precision and recall, we cannot directly evaluate the quality of the three similarity measures based on the reference clusters. The three similarity measures were selected based on our previous experience and we observed that they all contributed to the distance function for clustering. However, there exist quite a lot of similarity measures and some of them may be effective as well. Systematically comparing them will be one of our future work.

## Conclusion

We designed cutCluster, a scalable agglomerative clustering algorithm to address data quality issues in biomedical metadata. We have shown that our approach works especially in those cases when it is unknown how many different categories are present and also when there is no gold standard available. We selected a total of 359 keys from the GEO dataset for this experiment. These keys were chosen based on six characteristics (categories of keys): (i) age, (ii) cell line, (iii) disease, (iv) strain, (v) tissue and (vi) treatment. We manually created a gold standard to compare our results against which consisted of 27 clusters. By using cutCluster, 18 clusters were generated, containing all the 355 GEO keys (four clusters with only one key were excluded). In the 18 clusters, there were 342 keys that were identified correctly to be related to that cluster and thus similar to one another, but 13 keys were identified which were not related. Our algorithm identified keys that were similar based on (i) name, (ii) core concept and (iii) value similarities, to each other and grouped them together. Our algorithm also performed better than four other clustering algorithms. Also, we showed that our methodology is applicable to other keys and scalable. By using this method of clustering similar keys together, we are able to find keys which are related to each other even if they use different terms as well as duplicate and inconsistent keys in the dataset. That is, since metadata keys which are similar to one another are in one cluster, this helps address the problem for researchers to find related studies using a particular metadata keyword. This in turn will enable researchers to perform meta-analysis and systematic reviews using the GEO dataset.

As future work, we intend to improve our algorithm by more sophisticated measures for term similarity and cluster distance in order to help detect even further smaller clusters of related keys. Also, we plan to extend our work to find clusters of similar values. Moreover, after detecting the similar key-value pairs, we aim to use crowdsourcing methodologies to help verify, find and potentially fix these quality issues, especially those that can be easily detected by humans but not by machines. Ultimately, we aim to make the clean metadata values available directly with the existing GEO data to help enhance the re-usability of the dataset.

## Endnotes

^1^ Statistics derived from http://www.ncbi.nlm.nih.gov/geo/, Last accessed June 20, 2016.

^2^ Available at http://gbnci.abcc.ncifcrf.gov/geo/index.php (version January 23, 2016, 07:23:09, 264.5 MB).

## Abbreviations

GEO: Gene expression omnibus
MIAME: Minimum information about a microarray experiment
